# Deep learning course development and evaluation of artificial intelligence in vocational senior high schools

**DOI:** 10.3389/fpsyg.2022.965926

**Published:** 2022-09-23

**Authors:** Chih-Cheng Tsai, Chih-Chao Chung, Yuh-Ming Cheng, Shi-Jer Lou

**Affiliations:** ^1^Department of Industrial Technology Education, National Kaohsiung Normal University, Kaohsiung, Taiwan; ^2^General Research Service Center, National Pingtung University of Science and Technology, Pingtung, Taiwan; ^3^Department of Computer Science and Information Engineering, Shu-Te University, Kaohsiung, Taiwan; ^4^Graduate Institute of Technological and Vocational Education, National Pingtung University of Science and Technology, Pingtung, Taiwan

**Keywords:** deep learning, artificial intelligence, small private online courses, vocational senior high school, education reforms

## Abstract

This study aimed to develop cross-domain deep learning courses of artificial intelligence in vocational senior high schools and explore its impact on students’ learning effects. It initially adopted a literature review to develop a cross-domain SPOC-AIoT Course with SPOC (small private online courses) and the Double Diamond 4D model in vocational senior high schools. Afterward, it adopted participatory action research (PAR) and a questionnaire survey and conducted analyses on the various aspects of the technology acceptance model by SmartPLS. Further, this study explored the impact on the effects of deep learning and knowledge-ability learning of artificial intelligence after 16 weeks of course teaching among 36 Grade I students from the electrical and electronic group of a vocational senior high school. This study revealed that (1) the four stages of the SPOC-AIoT Teaching Mode of the Double Diamond 4D model may effectively guide students to learn AIoT knowledge and skills. (2) Based on the technology acceptance model, the analysis of learning and participation in SmartPLS indicated that this model conformed to the academic fitness requirements of the overall model. (3) After learning with the SPOC-AIoT Teaching Mode, the learning effects of students in AIoT have been significantly improved to a positive aspect. Finally, some suggestions were put forward to promote the development of the SPOC-AIoT Teaching Mode Course in the future.

## Introduction

To cultivate the key skills of future talents in learning, innovation, and digital literacy and simplify their lives and work, various countries have listed the “programming language” as a must-learn course in information technology education, which has driven a craze for national programming as well. The innovation of science and technology has accelerated globalization, narrowing the proximity among people and changing the speed and mode of learning. Various countries are also developing artificial intelligence (AI) to improve competitiveness, in which programming language is an entry ticket. Therefore, it can be noted that programming language is a basic skill and key ability in information technology. It is considered not only the most important second language in the world but also a significant technology education course.

In response to this wave of AI and programming, education reforms in various countries focus on cultivating students to be innovative talents in the twenty-first century, incorporating AI, the deep learning of AI, computational thinking, and the Internet of Things into the curriculum. In addition to building students’ information literacy and application ability, education reforms also cultivate their ability to think creatively and solve problems by teaching information technology. Further, “computational thinking” plays a significant role in learning and employment—some countries, such as the United States, United Kingdom, Germany, and Australia, have also listed information education as the focus of education reforms. In 2019, Taiwan incorporated programming language into the computational thinking course in the technology field in vocational senior high schools, allowing students to understand the principle of computational thinking for further interdisciplinary integration and applications—thus, having basic programming skills and key abilities. To face the upcoming challenge of Industry 4.0, the Internet of Things and AI have become key disciplines for emerging technologies. Furthermore, many scholars pointed out that vocational senior high schools should arrange courses that focus on students’ needs or industry trends to allow them to apply their professional knowledge to work and enhance their employment competitiveness (Tsai et al., 2019).

After reviewing relevant literature, the programming teaching in domestic and international vocational senior high schools takes the Maker Movement or STEAM as the framework and applies Scratch programming as a tool to probe into the framework’s teaching effect. Meanwhile, to implement “computational thinking” and “maker spirit” and consider the future movement of students, this study took AI deep learning to carry out AIoT Courses in the technology field of vocational senior high schools. It also took Python language as the course content to shift the focus of course arrangement and teaching through the Double Diamond 4D Model of dual divergence and convergence processes, such as problem clarification and definition and method formulation and practice. Furthermore, this study applied SPOC flip teaching from turtle graphics to the practices of Python and the implementation of learning transfer of computational thinking. Through the practical operation and application of AIoT courses, students’ learning and ability in “doing, using, and thinking” was realized. Moreover, it is expected that the course can be vertically linked with the emerging technology courses in colleges and horizontally linked with industrial trends to equip students with key abilities as innovative talents. Therefore, the purposes of this study are as follows:

(1) Build the development model of the SPOC-AIoT Model Courses in vocational senior high schools.
(2) Explore the applicability of the SPOC-AIoT Learning Scale in vocational senior high schools.
(3) Explore the learning effects of the SPOC-AIoT Model Courses in vocational senior high schools.

## Literature review

This study aimed to develop cross-domain deep learning courses of AI in vocational senior high schools and explore the effects of the course implementation.

Relevant explorations of literature are as follows:

### Application of the double diamond 4D model

The Double Diamond 4D Model, proposed by the British Design Council in 2005, is a prominent design process model, including four stages of exploration, definition, development, and realization. Emphasizing “divergent views” and “convergent thinking,” the model generates many ideas and then refines and narrows them into the best ideas. “Divergent views” and “convergent thinking” appear twice in this model; one confirms the definition of the problem, and the other creates a solution. The Double Diamond 4D Model illustrates four stages between two adjacent diamonds, each of which has the characteristics of fusion or conflict of thinking. (1) Discover problems—recognize the behavior patterns and problems facing the real world, (2) Define problems—after realizing the actual environment in use and encountering problems, record and determine the priority of the problems to “define the problems to be solved,” (3) Develop problem solutions—evaluate various solutions to determine the best method for achievement, and (4) Deliver solution selection and development—the final improved solutions.

Furthermore, AI has become widely used in design processes. To promote AI technology to effectively assist and meet the design needs of non-professional designers, Xu et al. (2020) started from the expansion stage of students’ thinking and employed the “Double Diamond Design Model” to provide unlimited possibilities for design tools in the future. In response to the diversity of students, D’Ettole et al. (2020) designed a cross-platform service blueprint based on the Double Diamond Design Process Model to solve various challenges faced by college students. Hence, this study on an AIoT characteristic course in vocational senior high schools took the Double Diamond 4D Model (Discover–Define–Develop–Deliver) as the framework of the course and teaching design. It guided students in learning through the four stages of Discover–Define–Develop–Deliver and responded to the current trend of AIoT applications while developing from practical operations.

### The impact and application of artificial intelligence on education

Artificial intelligence (AI) can be traced back to the Turing machine designed by Alan Turing in 1936. It is the prototype of the most important modern computer logic in computer science (Czerkawski and Lyman, 2015). AI has become an emerging technology along with computer software and hardware. Various countries are making plans to solve the application and impact of AI. Studies indicate that more innovative tools, intelligent applications, and methods are changing the education system, improving learners’ experiences and classroom learning results (Aoun, 2017; Papadopoulos et al., 2020). While studying the application evaluation of AI in medical education, Chan and Zary (2019) found that AI was applied to learning support, student evaluation, and course review; AI also provided students with guiding learning approaches and personalized feedback (Khumrina et al., 2018) or analyzed students’ academic performances through the neural network method to enable teachers to conduct more representative evaluation on students’ knowledge according to the course (Petrovskaya et al., 2020).

Artificial intelligence (AI) has thrived since its emergence. It plays a vital role in daily life, drastically changing our mindsets, behaviors, and interaction patterns, especially with the development of artificial neural networks (ANN) and deep learning (DL) (Chen et al., 2020). The working principle of DL is to process information like a human brain to strengthen neural networks through several hidden layers and to improve predictive ability (Feuerriegel and Fehrer, 2016). Akanbi et al. (2020) indicated that DL contains complicated structures and connections in the high-dimensional data and can represent new attributes of task-specific dynamic construction through data (Wan et al., 2014). This enables the deep learning model to outperform existing machine learning methods (Mayr et al., 2016). For instance, DL has been applied to computer vision and image processing (Pang et al., 2017), speech recognition (Fayek et al., 2017), traffic control (Zhao et al., 2017), electric power and energy consumption (Fan et al., 2017), business credit scoring (Luo et al., 2017), molecular analysis of medication (Ma et al., 2015), management of building energy consumption (Wei et al., 2020), natural language processing (Costa-jussà et al., 2017), and interpretation of medical images (Sufian et al., 2020).

With the great leap in information technology, on the one hand, the maturity of AI technology can finally be achieved—a new opportunity paving the way for creating competitive advantages among enterprises. On the other hand, the education field has also offered the opportunity for computer teaching, further promoting educational reforms and teaching processes. Hwang et al. (2020) pointed out that combining AI and education has greatly improved teaching quality and developed new teaching strategies. Teachers have benefited from the evaluation and data of the smart system, the improvement of students’ learning, and the development of new teaching strategies. Meanwhile, students have also improved learning effects from smart tutors and asynchronous learning. Therefore, the combination of AI and education is not only a change in education but also a breakthrough in human knowledge, cognition, and culture. Hence, the development of course models in this study focused on AI and the Internet of Things.

### Technology acceptance model

Among the theories of new technology products or innovative services, the most extensive is the Technology Acceptance Model (TAM) by Davis (1989). According to the review of 42 systematic evaluations of digital learning conducted by Šumak et al. (2011), it was found that the Technology Acceptance Model (TAM) was the most popular theory in digital learning, of which 86% considered “Technology Acceptance Model” as a reference (Al-Fraihat et al., 2020). Studies have also verified that the “perceived usefulness” and the “perceived ease-of-use” had a direct effect on learners’ will to use digital learning (Calisir et al., 2014; Hsia et al., 2014; Tarhini et al., 2014; Al-Gahtani, 2016; Chih-Cheng et al., 2019).

Technology Acceptance Model (TAM) has been widely used in various fields (Khan et al., 2021). Many other models have also been developed, such as the Theory of Reasoned Action (TRA), Innovation Diffusion Theory (IDT), and Unified Theory of Acceptance and Use of Technology (UTAUT). UTAUT was proposed by Venkatesh et al. (2003). It was integrated by the key factors of eight theoretical models, including TRA, TAM, Motivational Model (MM), Theory of Planning Behavior (TPB), Combined TAM and TPB (C-TAM-TPB), Model of PC Utilization (MPCU), IDT, and Social Cognitive Theory (SCT). The variables of this integrated model include performance expectancy (PE), effort expectancy (EE), social influence (SI), facilitating conditions (FC), behavior intention, behavior, gender, age, experience, and voluntariness of use. Rondan-Cataluña et al. (2015) used non-linear relations with more explanatory power than traditional linear methods. Alomary and Woollard (2015) confirmed the basic theories and their potential applications. They concluded that TAM laid a foundation for further understanding of the relationship among pedagogy, technology, and epistemology in the use and development of technologies.

Shen and Chuang (2010) pointed out that while verifying students’ attitudes and behavioral intentions by the extended Technology Acceptance Model with interactivity and self-efficacy, it was found that attitudes and behavioral intentions were affected by interactivity, self-efficacy, usability, and perceived usefulness. Abdullah and Ward (2016) pointed out that the Technology Acceptance Model was most affected by five external factors: self-efficacy, subjective norm, enjoyment, computer anxiety, and previous experience.

This study applied the mixed method involving face-to-face learning in traditional classrooms and online digital learning and built the teaching strategy using the Double Diamond 4D Design to conduct AIoT practical course teaching for students from vocational senior high schools. It also took the Technology Acceptance Model as the theory and self-efficacy and learning anxiety as external factors to explore students’ learning effects and learning satisfaction after taking AIoT courses.

## Research method

This study explored the basis for developing AI and the Internet of Things (AIoT) courses. Its relevant research method and design are as follows:

### Research structure

**FIGURE 1:**
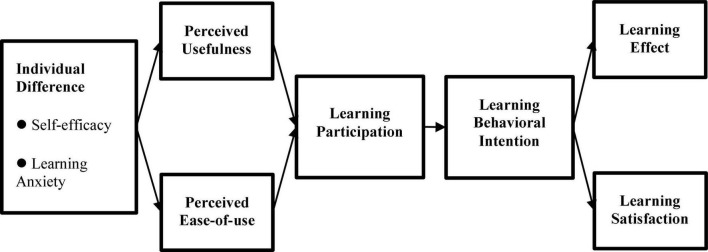
Research structure chart.

This study took participatory action research (PAR) as the principal axis to probe into the effects of “AI + Internet of Things (AIoT)” courses for students of vocational senior high schools, as shown in Figure 1. Based on small private online courses (SPOC) and through the design of the Double Diamond 4D Model (the Double Diamond Model), this study made learners the center of learning when developing AIoT courses. According to literature and studies, operational definitions of all dimensions are arranged in Table 1 of this study. Qualitative and quantitative analyses were integrated to recognize the learning effects and satisfaction degree in teaching activities. Further, the SPOC-AIoT Teaching Model and characteristic courses were built.

**TABLE 1:** Operational definitions of all dimensions in the SPOC-AIoT learning scale.

Name of variable	Operational definition
Self-efficacy	Learners’ cognitive confidence in computers and the Internet-related capabilities and knowledge learning of AIoT.
Learning anxiety	Learners are generally uneasy, worried, or afraid when they need to learn about AIoT *via* computers at present or in the future.
Perceived ease of use	How easy is it to use technologies do learners think after they learn AIoT?
Perceived usefulness	How much performance will be improved, or how many efforts will be spared? Do learners believe in learning AIoT through technologies?
Learning engagement	The process in which learners make consistent efforts to achieve the goal of learning AIoT.
Behavioral intention	How strongly are learners willing to study AIoT through information systems?
Learning outcomes	The knowledge acquired or capabilities demonstrated by learners when they finish the AIoT course or get their degrees.
Learning satisfaction	How satisfied or happy are learners in studying AIoT when they obtain teaching services in every aspect?

### Research subjects

This study took 36 Grade I students from the electrical engineering and electronic engineering group in a vocational senior high school in Taiwan as subjects. The course lasted 16 weeks in total with 2 classes per week. Apart from collecting students’ learning lists of experimental courses, this study performed the Information Technology Course Knowledge test before and after the experiments to compare students’ knowledge and ability differences in computational thinking, programming language, and AIoT. Finally, the SPOC-AIoT Learning Scaling questionnaire was implemented to examine the interrelation of self-efficacy, learning anxiety and effects, and learning satisfaction after AIoT courses.

### Research tools

The questionnaire of this study belonged to a self-report inventory. To avoid error variation in measurement due to the same source bias, this study took the advice put forward by Podsakoff et al. (2003) on the arrangement of the questionnaire. It adopted preventive measures such as hiding information on respondents and meanings of items, randomly allocating items, and rephrasing items. Such methods were required to minimize the error caused by common method variance (CMV).

(1) Regarding evaluating and figuring out collinearity among all variables, this study assessed it using the Variance Inflation Factor (VIF). Hair et al. (1998) state that collinearity exists when VIF values are greater than 5. Items with VIF values more than 5 were deleted after analysis, including la_2, bi_1, bi_3, lo_2, lo_5, le_3, ls_1, ls_5, peu_1, peu_2, and pu_2. The VIF values of the remaining items were between 1.152 and 3.805, which was within the recommended range (Hair et al.). As a result, the models of this study had no collinearity, as shown in Table 2.

**TABLE 2:** The validity analysis of each structure from SPOC-AIoT learning scaling.

Structure	Item	Factor loading (out loading)	Cronbach’s α	CR	AVE
Learning anxiety	la_1	0.913	0.782	0.901	0.821
	la_4	0.898			
Behavioral intention	bi_2	0.889	0.883	0.886	0.795
	bi 5	0.894			
Learning effect	lo 1	0.931	0.756	0.858	0671
	lo_3	0.795			
	lo_6	0.717			
Learning participation	lp 1	0.722	0.679	0.805	0.509
	lp_2	0.733			
	lp_4	0.712			
	lp_6	0.684			
Learning satisfaction	ls 2	0.808	0.815	0891	0.732
	ls_3	0.926			
	ls_6	0.829			
Perceived usefulness	peu 3	0.861	0.723	0.844	0.644
	peu_4	0.818			
	peu_5	0.722			
Perceived ease-of-use	pu 1	0.820	0.750	0.856	0.666
	pu_4	0.781			
	pu_5	0.845			
Self-efficacy	se 1	0.893	0.883	0.911	0.632
	se_2	0.745			
	se_3	0.855			
	se_4	0.787			
	se_5	0.725			
	se_6	0.748			

To recognize the learning effects and satisfaction degree of AIoT courses, this study prepared research tools of the Test Scaling in Information Technology Course Knowledge and SPOC-AIoT Learning Scaling. Experts and scholars were invited to review the scaling content to establish expert validity. In terms of reliability, as shown in Table 2, α value of each structure lies in the range of 0.679–0.883, beyond the lowest threshold value, belonging to medium–high reliability. In terms of composite reliability, the CR value of each structure lies in the range of 0.805–0.911, higher than the threshold value of 0.7 (Chin, 1998). Based on the factor loading of 0.7 as the threshold value (Hair et al., 1998), items la_3, la_6, bi_4, ls_4, lo_4, lp_5, and pu_3 were deleted in terms of index reliability. However, Item lp_6 (0.684) was kept considering dual factors of general reliability index and index reliability. The range of factor loading for the remaining items was between 0.712 and 0.931. Therefore, this questionnaire had good reliability. The smart-home homework list, the AIoT learning list of practical operation, the assessment scaling in the file report of learning processes, and the assessment scaling in the oral report of special topic production were utilized as auxiliary materials for students in the learning processes.

(2) Regarding construct validity analysis, convergent validity demands a consistency of all dimensions. Average variance extracted (AVE) values of all dimensions checked in this study were between 0.509 and 0.821, higher than the recommended threshold value of 0.5 (Fornell and Larcker, 1981). This shows that the average explanatory powers of all dimensions to indexes were over 50%, indicating convergent validity.

(3) Regarding discriminant validity, one of the prerequisites to analyzing structural models is to test such validity of different dimensions. The cross-loadings and Fornell–Larcker criterion were mostly used in assessing discriminant validity. As shown in Table 3, all dimensions’ factor loadings were greater than the cross-loadings between the dimensions and others. According to the Fornell–Larcker criterion, the discriminant validity is established if the square roots of AVE of all dimensions were more than the coefficients of correlation between the dimensions and the others. The results of this study showed that the square roots of AVE of all dimensions were between 0.713 and 0.906. They were more significant than the correlation coefficients between those and the other dimensions. As shown in Table 4, convergent validity was achieved in all dimensions. Thus, it was proved that all dimensions in this study have construct validity.

**TABLE 3:** Cross-loadings of all dimensions in the SPOC-AIOT scale.

Structure	Learning anxiety	Behavioral intention	Learning effect	Learning participation	Learning satisfaction	Perceived usefulness	Perceived ease-of-use	Self-efficacy
la_1	0.913	–0.386	–0.316	–0.270	–0.384	–0.169	–0.147	–0.249
la_4	0.898	–0.255	–0.370	–0.211	–0.324	–0.179	–0.106	–0.025
bi_2	–0.302	0.889	0.522	0.535	0.698	0.397	0.596	0.488
bi_5	–0.333	0.894	0.450	0.690	0.646	0.376	0.564	0.539
lo_1	–0.431	0.581	0.931	0.527	0.655	0.334	0.458	0.518
lo_3	–0.306	0.397	0.795	0.335	0.543	0.024	0.366	0.181
lo_6	–0.102	0.289	0.717	0.368	0.318	0.438	0.357	0.308
lp _1	–0.279	0.481	0.526	0.722	0.498	0.359	0.417	0.393
lp _2	–0.095	0.582	0.252	0.733	0.467	0.301	0.557	0.259
lp _4	–0.205	0.477	0.309	0.712	0.463	0.565	0.440	0.402
lp _6	–0.199	0.417	0.399	0.684	0.609	0.523	0.576	0.586
ls_2	–0.317	0.617	0.652	0.575	0.808	0.405	0.644	0.579
ls_3	–0.334	0.681	0.641	0.626	0.926	0.385	0.604	0.547
ls_6	–0.354	0.634	0.365	0.631	0.829	0.356	0.424	0.487
peu_3	–0.162	0.295	0.228	0.466	0.288	0.861	0.429	0.455
peu_4	–0.087	0.207	0.087	0.301	0.256	0.818	0.499	0.570
peu_5	–0.200	0.500	0.396	0.657	0.491	0.722	0.565	0.406
pu_1	–0.251	0.647	0.579	0.594	0.611	0.548	0.820	0.615
pu_4	0.002	0.364	0.164	0.484	0.424	0.432	0.781	0.514
pu_5	–0.074	0554	0.402	0.629	0.543	0.533	0.845	0.541
se_1	–0.326	0.524	0.417	0.508	0.646	0.490	0.565	0.893
se_2	0.074	0.326	0.122	0.381	0.305	0.537	0.480	0.745
se_3	–0.252	0.583	0.417	0.622	0.576	0.479	0.658	0.855
se_4	–0.072	0.447	0.386	0.440	0.500	0.470	0.662	0.787
se_5	–0.075	0.296	0.307	0.340	0.311	0.465	0.310	0.725
se_6	0.111	0.390	0.294	0.501	0.569	0.496	0.440	0.795

**TABLE 4:** Reliability analysis of all dimensions in the SPOC-AIOT scale.

Structure	Formell–Larcker							
	1	2	3	4	5	6	7	8
Learning participation	0.713							
Learning effect	0.513	0.819						
Learning satisfaction	0.714	0.647	0.856					
Learning anxiety	–0.267	–0.378	–0.392	0.906				
Self-efficacy	0.611	0.434	0.628	–0.156	0.795			
Behavioral intention	0.688	0.544	0.753	–0.357	0.576	0.892		
Perceived usefulness	0.612	0.311	0.446	–0.192	0.594	0.433	0.803	
Perceived ease-of-use	0.703	0.484	0.651	–0.141	0.684	0.651	0.631	0.816

### The development of the double diamond 4D design courses

Based on the four stages of Discover–Define–Develop–Deliver from the Double Diamond 4D Model, this study conducted teaching practice arrangement as follows:

#### Discover: Generate interest

This stage is to arouse learning motivation, resonating with students to actively participate in the course. This study applied cooperative group learning. Instead of directly introducing Python grammar and coding into the teaching operation, it adopted the practical operation of AIoT-Smart Home to build and enlighten the application of AI and the Internet of Things for students in the process of learning by doing.

Taking turtle graphics as the medium to inspire teaching activities and through the geometric graphics process, the course arrangement made students understand the basic objects and function instructions of the Python language. In addition, the concept of dummy coding was supplemented as an intuitive compilation and graphics step to help students understand the course content as fast as possible. Furthermore, it introduced the concept of programming language coding and repeated the original turtle graphics homework with loop grammar to motivate students. The development modules of this study had 17 units, such as turtle graphics, touch/RC module, a sensing module, voice recognition module, and image recognition module. Furthermore, the teaching team of this study shot teaching films, sorted out class materials, and uploaded them to the teaching website to allow students to watch and read online flexibly, as shown in Table 5. Sources of related films available online were also added for auxiliary learning.

**TABLE 5:** Courses of five modules.

Module course	Course unit	Course target
Turtle graphics	Introduce and install the Thonny editor Learn python for graphics Strengthen the thinking and process of programming with a program flow chart	Learn python objects, functions, and modules through the graphic process.
Touch/RC module	Introduce functions and the assembly of components of the Genio demo board Touch device teaching RC device teaching	Understand and learn to assemble the Genio demo board, and combine the Genio demo board, LED, and press switch. Test the signal output and LED on/off through the program press switch, and combine the mobile APP to conduct the RC of LED on/off.
Sensing module	Principle and demonstration of temperature and humidity sensors Analog temperature and humidity sensor element opening and closing LED (air conditioner) Principle and demonstration of ultrasonic sensors Analog ultrasound sensor element opening and closing LED (sensor light) Principle and demonstration of photosensitive resistor sensor Analog photosensitive resistance sensor element opening and closing LED (breathing lamp)	(1) Learn to use temperature and humidity sensors to detect environmental temperature and humidity, integrate the program codes to control the opening and closing of air conditioner and dehumidifier, and monitor the temperature and humidity at the same time to open and close LED1 (air conditioner) and LED2 (dehumidifier). (2) Learn the distance measurement function of ultrasonic sensors and simulate the parking sensor to detect distance and warning sound. (3) Learn the photosensitive resistor sensors and analog light-sensing devices for the application of opening and closing of LED (breathing lamp).
Voice recognition module	Explain the playback process and demonstration operations of voice recognition Play music by password	Learn the application of the audio_decode sound effect module and replace the built-in canned music. Import the voice recognition module with the voice recognition function and play the music through voice commands.
Image recognition module	Brief introduction to image recognition theory Face training, recognition, and supporting procedures Modify the graphic file of the boot screen	Understand the training process and prediction results of image recognition theory to clarify the recognition process. Modify the built-in graphic file of the boot screen of the demo board.

#### Define: Teaching activities

This stage is to focus on the teaching of basic function grammar coding. This study emphasized the learning process of students in teaching, supplemented by online videos and group-sharing rather than individual learning. Therefore, through the learning mode of dual roles both as learners and teaching scholars, students’ learning effects might be improved.

The online teaching website of this study has a function that analyzes login times and online time. Before teaching each module course, a preclass test would be initially carried out, and then the course discussion and practical operation teaching would proceed. In addition to the implementation of flip teaching, it is necessary to preview the teaching films to achieve self-learning. After evaluation in class, group module teaching and practical operation discussions were conducted to achieve learning effects.

#### Develop: Focus on learning

This stage not only continues the mixed teaching and group-sharing of the previous stage but also introduces experienced practitioners to guide students for practical operations, turning theory into practice and strengthening students’ concepts. The subjects of this experiment were students from the electrical and electronic group in vocational senior high schools. This course focused on AIoT applications, and smart homes were realized by controlling home environments. As a result, this study combined AI and Internet of Things concepts with applications of practical operations.

Apart from teaching practical operations with modules, the course also provided lectures on deep AI learning and utilizing big data to segment data and extract target and characteristic values. Then, it predicted the model trained with the remaining data after the algorithm training model and verified the prediction with practical operations of image recognition module courses to deepen students’ concepts. Besides instructing the theory of the Internet of Things, it also took the films of living examples applied to life as auxiliary teaching.

#### Deliver: Result demonstration

In this stage, in the process of assembling the teaching materials of various modules into a smart home, students were able to build their own learning experiences and processes regarding AIoT. The established online learning environment provided an opportunity for students to learn independently and enrich their professional knowledge and ability. Each group was scored based on creativity, program optimization, design concept, and visual beauty of their briefing. They would also show their achievements, such as individual acquisition and geometric graphics of the group (contrastive graphics and loop commands). Further, the scoring on the practical operations was based on functions and team cooperation.

## Results and discussion

### Analysis of students’ learning processes

This study applied the SPOC-AIoT Teaching Model to study, observe, and collect students’ feedback and demonstrated in the order of Discover–Define–Develop–Deliver as follows:

#### Discovery

(1) The course was carried through to the end, and the arrangement echoed the end with the beginning.

To improve students’ interests in learning and make them feel the utility and fun of the program, the course guided students to make graphics with Turtle graphics and presented the finished works on the LCD screen of a smart home as a substitute to the original settings. By doing so, this course aimed to improve students’ interests in learning programming language and participation in the course.

(2) The teaching model of each module should be consistent.

To reduce students’ learning burden and avoid weakening their confidence, the course arrangement of each module applied the same model. For instance, in the Turtle graphics unit, the intuitive code programming method was adopted for the students to familiarize themselves with Python grammar. The same example was used to rewrite in the loop or discriminant form. The learning list was used to conceive the geometric graphics that each group wanted to present to make students proficient in the loop or discriminant grammar. Further, the list was taken as a code programming mode for the course in each module in the future. In this way, it presented the internal thinking of students and exposed the thinking to the exterior by driving the hardware through the main board to shorten learning processes and focus on the grammar required by this course instead of learning the whole programming course.

(3) Recognize the program grammar rather than write the program.

This course involved learning AIoT-related knowledge, allowing students to experience AIoT through programming language and practical operations. It must also be noted that writing programs is not the purpose of teaching in this experiment. Therefore, in the course arrangement, sample program codes that could be copied were provided so that students could make use of them and conduct tests. With the understanding of the program code, they could transform the code into the program code to express personal thinking through modification. Therefore, the essence of this experiment was to make students focus on the grammatical structure rather than the input method.

#### Definition

There are many functions in the development edition of teaching in this experiment. Thus, it is hard to instruct entirely according to unit teaching. Meanwhile, students may fail to achieve outstanding learning effects because they cannot integrate the contents of each unit. Therefore, according to the functional attribute of parts/components, the course arrangement in this experiment was classified into five categories of module courses to simplify the teaching process, thus improving teaching efficiency.

The courses in this module consisted of five categories of courses, which were divided into 17 units with different module operation modes due to different mobile phone systems. The mobile phone system of teachers was Android, and students who owned Apple would be provided with online teaching films as auxiliary. During instructions, the touch/RC module modes of these two systems would also be specially illustrated.

The feedback from students is as follows:

• Unexpectedly, I have completed my professional practice and become interested in electronics. I want to challenge myself and learn more about how to use it (20210428).
• Whenever I saw these modules, they had already been assembled, and I had never viewed their internals nor understood their principles. By introducing these courses, I have learned a lot and understood more (20210210).
• I hope the program can be made as convenient for Apple as for Android phone users to learn. Although some unit courses are difficult, I believe that they will be definitely helpful in the future for me to study hard (20210530).

#### Development

**FIGURE 2:**
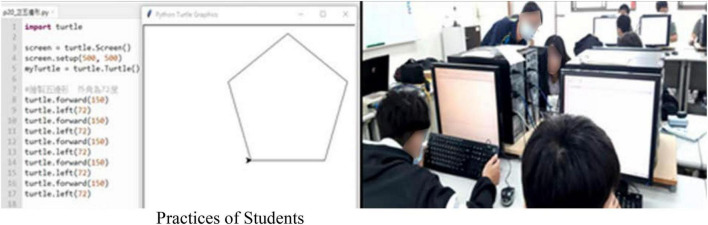
Graphics python loop.

The module courses arranged in this stage were combined with theory and practice to strengthen the knowledge and practical operation ability in the programming language, IoT-related sensors, and AI for students from the electrical and electronic group in vocational senior high schools. In this case, students may complete this study topic (i.e., smart home featured by AIoT’s application in the control of the home environment) and learn the application of AI and IoT concepts and practical operations. The implementation of the courses and the learning state of students are illustrated as follows:

In Python graphics, Python language has an open-source feature for the availability of many functional components. This study arranged students to systematically learn concepts, such as object-oriented concepts, component, and module importing methods and to explain the errors that are easy to make during python code writing. For instance, single quotation marks (“) or double quotation marks (“ ”) must be imported around string data to include string data; it is forbidden to use with mixed single and double quotation marks and impossible to directly add or subtract strings and numbers. In this way, students may quickly get into the learning state. When students had a python object-oriented basic coding concept, teachers would apply the Turtle graphics module coordinating with related functions to conduct graphics teaching, from practices of basic geometry graphics (such as quadrangle and pentagonal) to practices of advanced graphics (petal), as shown in Figure 2.

**FIGURE 3:**
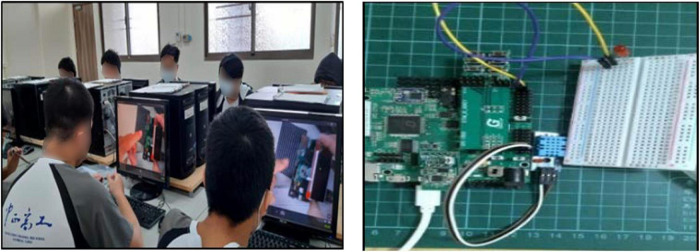
The practical operation of Genio Demo board.

In the practical operation of the Genio demo board, a Genio demo board teaching tool was used in all module courses. In particular, the course arrangement in this module introduced the names and functions of each component on the demo board, including the photography module, LED screen module, bluetooth module, and motherboard module of the demo board. Through the physical projector for teaching demonstration, each component was combined and used as the teaching tool for this unit, connected with the main frame, and checked whether the LED light was on to test whether the motherboard was abnormal. Subsequently, students were reminded of assembly points for attention in the process and made each group practice assembly and testing on their own, as shown in Figure 3.

**FIGURE 4:**
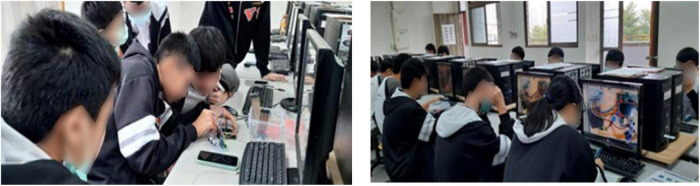
The practical operations and stimulating applications of the ultrasonic sensor.

**FIGURE 5:**
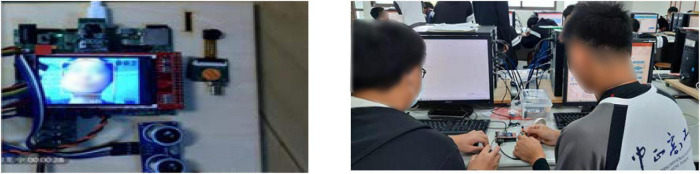
The practical operation practices of image recognition module.

In teaching each module, teaching strategies such as lecture teaching, demonstration, operation practices, and program writing and testing were employed from demonstration teaching of teachers to the practical operation activities of students, as shown in Figures 4, 5, along with program writing, update setting parameters, and observe the test value changes on the screen. For instance, the temperature and humidity sensor with a hairdryer was heated; when the temperature value detected was higher than 30°C, an LED lamp would shine. Further, students were instructed to move in front of the ultrasonic sensor with a pad to block; when the distance from the value detected was less than 20 cm, the LED lamp would shine. Then, the photosensitive resistor or flashlight by hand was covered to observe the value change on the screen. Finally, access control testing was simulated among team members in the image recognition course. Based on these, students might have gained deeper impressions to learn and understand the principles and operations of various sensing components, thus strengthening learning effects.

The arrangement of module courses in this study differs from other information technology courses in Grade I of vocational senior high schools. In addition to learning the AIoT concept, students were required to operate various modules by hand, complete various assignments, and finish the works of a smart home. It was difficult for students who had just studied in a vocational senior high school and had never come into contact with emerging information technology, especially in the practical operation courses. When students did not follow the teaching instructions and instead conducted the wrong step, this would result in errors in the module components.

Before conducting the course, the researcher of this study practiced continuously to be familiar with the operation of various modules to respond to emergencies. While assigning students tasks of various modules, future teachers should ensure that they have practiced in person. In addition to an actual demonstration in class, they would need to record teaching videos and upload them to the learning platform for students to watch after class. Furthermore, teachers should simplify various processes and module operations to make it easy for students to learn and control the time of practical operations, avoid affecting subsequent module courses, and maintain class order during students’ discussions of practical operations. They should also encourage students to make good use of the learning platform to communicate, share, and consult questions, and be ready to help students solve their doubts at any time.

The feedback from students is as follows:

• Although the information technology course is different from courses in other classes, through teaching and performing practical operations of AIoT courses, we can learn online anytime and anywhere without the limitation of time or place, making learning very convenient. This course is very suitable for long-distance teaching (20210134).
• When doing homework at home, if you do not know or are not sure about the operation process and are afraid of burning the circuit, you may view teaching films on the learning platform to finish the homework smoothly (20210119).
• With the assistance of teaching films, it will be helpful for innovation, motivating one to strive for perfection and design better-finished works to present various appearances (20210103).
• I have learned about the operation, principle, and running of smart control, like LED lights which can also be adjusted and modified according to our own methods (20210222).
• I have learned about the use of AIoT in life and also learned to use program codes to make machines operate, such as Red Line lights (20210231).
• Learning wiring and circuit board installation in advance is helpful for the practice and application of electronics, even laying the foundation of electronic courses (20210202).

#### Delivery

In this stage, students applied the knowledge and ability learned in each module course to show their learning achievements through the finished works of a smart home. The teaching focused on the learning and doing of students. The students were willing to learn emerging technologies, take the initiative to operate, and complete various tasks and smart homes at the end of the term as scheduled. Although the complete implementation program has been built into the memory card, this experimental teaching aimed to recognize and read the program. Therefore, in the program implementation, each group modified the judgment parameter, background music or LCD screen patterns, and characters for each sensor, all of which presented their learning effects.

The contents of the courses were mainly for students, most of whom had been introduced to AIoT courses for the first time. In addition to teaching the concept of AIoT, this course also included program design and practical operation courses. Further, the course design replaced hard and difficult features with simple and interesting ones to arouse students’ learning and motivations. The teaching method employed flip teaching, characterized by lively and vivid activities, with students as the main body. The contents of this experimental teaching adopted theory and practical operation as equal emphasis, made use of practical operation to deepen the understanding of the theory, and applied the auxiliary teaching tools developed by manufacturers. Adopting a simplified process and high product stability made it easy for students to complete the final works and improved the degree of participation and the sense of achievement in the course.

Finally, the sharing of students was added into the process of this experimental teaching, with discussion and finished work displayed, making AIoT courses richer and more creative. Through discussions and sharing of each group, students learned AIoT from the concept establishment and task completion to the smart home–finished works display, complementing each other. The students were able to apply their learnings to stir up more creative ideas and finished works, further expanding their perception of AIoT courses in vocational senior high schools to start an extraordinary AIoT learning journey.

The feedback from students is as follows:

• Through flip teaching, the learning gets more diversified, and the course content is quickly understood. The class becomes much more interesting, and the teaching and learning effects are improved (20210403).
• We may have more learning space, motivating us to work harder to finish tasks. We may discuss with students online when encountering problems (20210419).
• If you do not understand the course, you may figure out what you do not understand on your own. Besides, there are groups, and you may directly consult the teacher (20210427).
• It is close to daily study, improves and draws materials from life, and inspires us to make the world more convenient in the future and connect various daily necessities with the Internet of Things (20210203).
• It improves our understanding of the Internet of Things, deep learning, and the operation of AI (20210233).
• “AIoT-smart home” simulates intelligent control, which not only makes me understand the importance of AI but also perceive the strength of AI. Therefore, AIoT learning is even more indispensable (20210206).

### Small private online courses-artificial intelligence and the internet of things teaching structure model analysis

**FIGURE 6:**
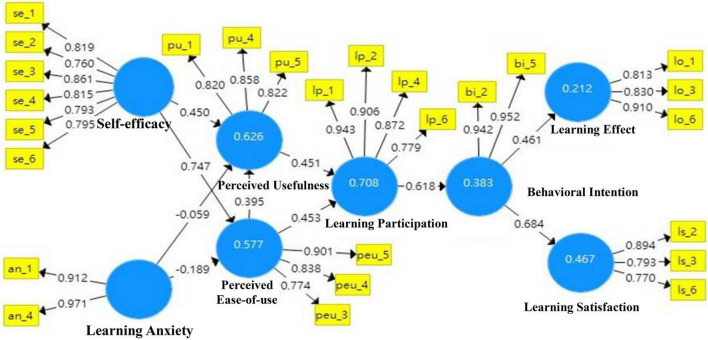
The entire model of SPOC-AIoT scaling.

This study adopted SmartPLS3 to make path analysis among various structures in the research structure. Through Bootstrapping, the path analysis of data was made in a repeated sampling method (5,000 times), and the research hypothesis was verified. Given that the research structure is a single clear directional relationship, this study used a two-tailed test with a significant level (*p*-value) of less than 0.05 as the judgment standard (Bassellier and Benbasat, 2004; Anuwichanont, 2010). The results of the entire model after statistical verification are shown in Figure 6.

According to Hair et al. (2014), a systematic method was proposed to evaluate the structure model. The process was divided into several stages, including multicollinearity diagnostics of the structure model, significance verification of the path coefficient, evaluation of *R*^2^ size, and the evaluation and explanation of the size of effect *f*^2^. Through analysis, after the items la_2, bi_1, bi_3, lo_2, lo_5, lp_3, ls_1, ls_5, peu_1, peu_2, pu_2, whose VIF value is greater than 5, were deleted, the VIF value of each index of the structure model was less than 5. Furthermore, the VIF values among relevant structures were less than the threshold value 5, as seen from inner VIF values, indicating that each index in the measurement and structure models and the multicollinearity problem among various structures had not reached the severity level (Hair et al., 2011). Thus, the multicollinearity problem in the future would not have adverse effects on the path coefficient estimation of the structure model. The test results are shown in Table 6.

**TABLE 6:** SPOC-AIoT scaling structure model VIF verification table.

Structure	Item	Variance inflation factor (VIF)	Inner VIF values							
			1	2	3	4	5	6	7	8
Learning anxiety	la_1	2.632							1.004	
	la_4	2.632								
Behavioral intention	bi_2	2.722			1.000	1.000				
	bi_5	2.722								
Learning effect	lo_1	1.583								
	lo_3	1.922								
	lo_6	2.136								
Learning participation	lp_1	4.804							2.168	
	lp _2	3.817								
	lp _4	3.099								
	lp _6	1.855								
Learning satisfaction	ls_2	2.054		1.000						
	ls_3	1.874								
	ls_6	1.312								
Perceived ease-of-use	peu_3	1.432		1.004					2.361	
	peu_4	1.965								
	peu_5	2.331								
Perceived usefulness	pu_1	1.594	1.008							2.321
	pu_4	1.689								
	pu_5	1.582								
Self-efficacy	se_1	2.312								
	se_2	2.179								
	se_3	2.752								
	se_4	2.575								
	se_5	2.129								
	se_6	2.634								

The testing results for significance verification of the path coefficient, evaluation of *R*^2^ size, and the evaluation and explanation of the size of effect *f*^2^ are sorted out, as shown in Table 7.

**TABLE 7:** The assessment and verification sheet for SPOC-AIoT scaling structure model.

Hypothesis	Relation	Path coefficient	*T-*value	Conclusion	*R* ^2^	*f* ^2^	Fitness
H1	Self-efficacy to perceived ease-of-use	0.747*	9.123	True	0.577	1.313	SRMR = 0.099 NFI = 0.487 RMS_theta = 0.250
H2	Learning anxiety to perceived ease-of-use	–0.189	1.482	False		0.084	
H3	Self-efficacy to perceived usefulness	0.450*	1.934	True	0.626	0.233	
H4	Learning anxiety to perceived usefulness	–0.059	0.441	False		0.009	
H5	Perceived ease-of-use to perceived usefulness	0.395*	1.889	True		0.176	
H6	Perceived ease-of-use to learning participation	0.453*	2.654	True	0.708	0.325	
H7	Perceived usefulness to learning participation	0.541*	2.666	True		0.322	
H8	Learning participation to behavioral intention	0.618*	5.432	True	0.383	0.619	
H9	Behavioral intention to learning effect	0.461*	2.460	True	0.212	0.269	
H10	Behavioral intention to learning Satisfaction	0.684*	11.569	True	0.467	0.877	

*Indicates that it is significant at a significant level of 0.05.

#### Verification of path relationship

In this study, the model’s path coefficients of the hypothesis relations of H1, H3, H5, H6, H7, H8, H9, and H10 are 0.747, 0.450, 0.395, 0.453, 0.541, 0.618, 0.461, and 0.684, respectively. All path coefficients reached significant levels, and the hypotheses were verified.

(1) Self-efficacy based on the Technology Acceptance Model, and the impact of implementing SPOC-AIoT teaching on learning effects and learning satisfaction.

In terms of self-efficacy, the AIoT flip course teaching could improve students’ learning effects. In other words, the higher the self-cognition confidence of students is in information and communication-related abilities and knowledge, the more helpful it is for digital learning effects. It can be seen from the qualitative analysis that students’ self-learning was no longer limited by time and space when they used the films and teaching materials on the teaching website. Not only that they showed repeated learning but they also studied deeply. Noteworthy, they focused more on films compared with books.

The mixed method involving face-to-face learning in traditional classrooms and online digital learning to conduct SPOC-AIoT flip courses improved students’ learning satisfaction. In other words, the higher the cognition of students to study through various learning channels, the higher their learning satisfaction will be. In particular, it can be seen from the qualitative analysis that students preferred online learning together with traditional teaching since online learning was convenient and interesting, which made learning easier. Further, it is worth noting that teachers complied with students rather than the other way around.

(2) Learning anxiety based on the Technology Acceptance Model—the impact of implementing SPOC-AIoT flip teaching on learning effects and learning satisfaction.

It can be seen from the quantitative results that learning anxiety had no significant impact on perceived usefulness and perceived ease-of-use in Technology Acceptance Model. In other words, no anxiety was generated in the process of students’ using digital technology to learn new knowledge. The analysis indicated that this might be because the arrangement of this experimental course summarized courses of the same nature into a module course to adopt systematic steps so that students can be familiar with its teaching pace. Furthermore, it provided detailed teaching assistance. For instance, teachers first filmed the practical operation process of each module course for students to preview before class and review after class. Although it was flip teaching, it took teaching in traditional classrooms and acquisition sharing of the group as auxiliaries, greatly reducing the learning anxiety of students. Therefore, through Technology Acceptance Model, this mechanism strengthened students’ learning effects and satisfaction in the flip learning model. The qualitative analysis indicates that no anxiety was generated when students used digital technology to learn new knowledge because online films could be viewed repeatedly without space limitations, and students could be online to learn at any time.

(3) Learning participation based on the Technology Acceptance Model—the impact of implementing SPOC-AIoT teaching on learning effects and learning satisfaction.

The results of quantitative research show that learning participation had a significant impact on perceived usefulness and perceived ease-of-use in the Technology Acceptance Model. Furthermore, it affected students’ attitudes and cognition when using communication technology to help their learning. In other words, digital technology was positive in learning new knowledge, helpful for self-learning, and easy to use, which did not cause any learning burden. Therefore, students actively put themselves into the learning state of the course, and the willingness to use information technology tools as auxiliary learning was improved, further improving their learning effects and satisfaction. According to the qualitative analysis, students stated that the reason why digital technology affected learning participation was that the actual operation, practices, and experiences made it easier to understand the basic theory, application, and convenience of AIoT, not only arousing their learning interests but also generating motivation to learn AIoT.

#### Assessment of explanatory ability

Generally speaking, when the *R*^2^-value is close to 0.25, it can be regarded as a little weak explanatory ability; when the *R*^2^-value is close to 0.50, the model has a medium explanatory ability; when the *R*^2^-value is close to 0.75, the model has a significant explanatory ability (Hair et al., 2014). According to this standard, the learning effect structure (0.212) in this research model has a weak explanatory ability—the behavioral intention structure (0.383) and the learning satisfaction structure (0.467) have a medium explanatory ability, while perceived ease-of-use (0.577), perceived usefulness (0.626), and learning participation (0.708) have high explanatory ability, as shown in Table 7.

According to Cohen (1988), the *f*^2^-value is proposed to evaluate whether exogenous variables had a significant explanatory ability on endogenous variables. The principle is as follows: 0.02 < *f*^2^ ≦ 0.15 is considered small effect, 0.15 < *f*^2^ ≦ 0.35 is considered medium effect, and *f*^2^ > 0.35 is considered large effect. According to the research results, self-efficacy to perceived ease-of-use (1.313), behavioral intention to learning satisfaction (0.877), and learning participation to behavioral intention (0.619) all have a high explanatory ability; perceived ease-of-use to learning participation (0.325), perceived usefulness to learning participation (0.322), behavioral intention to learning effects (0.269), self-efficacy to perceived usefulness (0.233), and perceived ease-of-use to perceived usefulness (0.176) have a medium explanatory ability; learning anxiety to perceived ease-of-use (0.084) and learning anxiety to perceived usefulness (0.009) have a weak explanatory ability. Overall, exogenous structures such as self-efficacy and learning anxiety had an above-medium to the highest explanatory ability to endogenous structure in Technology Acceptance Model, as shown in Table 7.

#### Hypothesis verification

The test results of the model in this study are SRMR = 0.099 > 0.08 and RMS_theta = 0.250 > 0.12, as shown in Table 7. Although the overall model fitness was not appropriate, the model fitness state in PLS-SEM should not only be determined by the fitness results but also be comprehensively evaluated by referring to the significance of the path coefficient and the model’s explanatory and prediction ability. Therefore, this model should conform to academic fitness requirements for the overall model.

### The analysis of small private online courses- artificial intelligence and the internet of things teaching model effect of students on artificial intelligence and the internet of things conceptual learning of information technology

This experimental teaching carried out AIoT conceptual knowledge tests before and after the module course teaching and adopted the verification of sample *t*-test to analyze the learning effects of students on AIoT conceptual knowledge of information technology. This study analyzed the aspects of computational thinking, programming language, AI concept, and IoT concept, as shown in Table 8.

**TABLE 8:** The verification and analysis of Students’ AIoT Knowledge of Information Technology on sample *t*-test.

Structure	Average	*SD*	*t*	*df*	Significance (Two-tailed)
CT_pre–CT_pos	–0.08681	0.20663	–2.521	35	0.016
Py_pre–Py_pos	–0.11458	0.23788	–2.890	35	0.007
IoT_pre–IoT_pos	–0.18056	0.24357	–4.448	35	0.000
ai_pre–ai_pos	–0.10764	0.21989	–2.937	35	0.006

According to the verification results, the average difference value measured around calculation thinking is –0.08681, reaching significance (0.016). This finding indicates that implementing the SPOC-AIoT Teaching Model could help students significantly improve their knowledge of computational thinking learning. The average difference value measured around programming language is –0.11458, reaching significance (0.007). This finding indicates that implementing the SPOC-AIoT Teaching Model could help students significantly improve their knowledge of program design learning. The average difference value measured around the Internet of Things (IoT) is –0.18056, reaching significance (0.000). This finding indicates that implementing the SPOC-AIoT Teaching Model could help students significantly improve their knowledge of the Internet of Things (IoT) learning. Furthermore, the average difference value measured for AI is –0.10764, reaching significance (0.006). This finding indicates that implementing the SPOC-AIoT Teaching Model could help students significantly improve their knowledge of AI learning.

## Discussion

The current educational trend has treated students as the main subject in the learning process. Therefore, students’ future development and adaptabilities should be considered when planning a living technology-related curriculum, whether they plan to advance to college or work in the fourth industrial revolution.

This study adopted the double diamond 4D model to construct the SPOC-AIoT teaching module in four stages. These stages are: discovering, defining, developing, and delivering. (1) In discovering stage, special courses on AIoT should be created, and online learning and flipped teaching methods should be adopted to motivate students based on the industrial trend and present situation analyses. (2) In the defining stage, the students should be provided with systemic and convenient learning materials and experiential learning based on theme-based modules. (3) In the developing stage, optimizing the contents of educational websites and utilizing teaching aids with technology trends should help improve the course content and students’ focus. (4) In the delivering stage, students’ active attitudes toward knowledge learning should be demonstrated in practical courses in middle schools, showing students’ satisfaction with the curriculum. The results of this study indicate that those four stages effectively lead to active learning of the students and focus on studying and utilizing AIoT to improve learning effectiveness and obtain higher satisfaction of students (Alomary and Woollard, 2015; Hwang et al., 2020).

MOOCs have been prevalent globally in recent years. The education system of a high school still differs from university. As a result, it was suggested in this study that innovative teaching should be conducted by combining traditional teacher–student interactions in classrooms and small private online courses (SPOC) with characteristics of flipped teaching methods such as MOOCs. Hence, students were provided with learning materials that were easy to understand and helped them learn better so that students’ learning effectiveness could be increased. Furthermore, they should realize the accessibility and usability of software and hardware and feel less anxious in the face of new knowledge or information literacy (Rondan-Cataluña et al., 2015).

Due to insufficient professional qualities in implementing experimental pedagogy, the researcher enriched and participated in various workshops to improve those qualities. As experts and scholars in various fields have pointed out, the main obstacle to promoting teaching new courses was that teachers could not achieve sustained growth. Consequently, the curriculum planning of all subject groups prioritized teacher expertise over student demands. This study could construct teaching modules for reference by cooperating with multidisciplinary experts to promote emerging technologies and foster capabilities in professional areas. However, whether such a model could be applied to other fields for improvements of expertise remains future work.

## Conclusion and suggestions

This study aimed to introduce the knowledge and ability of emerging technologies into vocational senior high schools to enable students to have the ability to link up with technical college courses or Industry 4.0 talents in the future. It also built a SPOC-AIoT Teaching Model suitable for vocational senior high schools to implement emerging technology courses. The subsequent results were verified through experimental teaching. The conclusion and suggestions are as follows:

### Conclusion

(1) This study built a SPOC-AIoT Teaching Model to effectively improve students’ learning of AIoT knowledge and ability.

According to the qualitative and quantitative analyses and the pretest and posttest results of “conceptual knowledge,” the cultivation of AIoT-related knowledge and abilities of students presented a significant improvement after students accepted the arranged courses based on the Double Diamond 4D Design Model and flip learning model. This finding indicates that the SPOC-AIoT Teaching model in this study effectively intensified students’ learning of AIoT conceptual knowledge and application ability.

(2) This study built a Technology Acceptance Model for the SPOC-AIoT Teaching Model, which helps analyze students’ learning effects and satisfaction.

After verifying and analyzing the applicability of the SPOC-AIoT Technology Acceptance Model Structure built in this study, it is shown that this study explored and analyzed students’ learning of AIoT-related knowledge and abilities by taking self-efficacy and learning anxiety as independent variables. In particular, the students’ learning participation based on the Technology Acceptance Model was helpful for learning effects and learning satisfaction.

(3) This study built a SPOC-AIoT Teaching Model to effectively improve students’ AIoT learning effects.

The experimental research of the SPOC-AIoT Teaching Model built in this study found that online learning was helpful for self-learning and convenient learning materials helped reduce the learning anxiety of students. Furthermore, learning participation with the flip teaching model helped improve learning effects and satisfaction. Therefore, this teaching module helped improve students’ learning effects and satisfaction with AIoT-related knowledge and ability.

### Suggestions

#### Normal development and promotion of SPOC-AIoT Teaching Model courses

When promoting the courses related to emerging technologies, schools organize the exploration activities of community type or short-term study most of the time. The learning effects and the number of participants are often limited, as well as the effects of deepening the literacy of emerging technology. It is suggested that schools develop cross-field cooperative learning normally and make it a regular course for each group of subjects to deepen students’ knowledge, ability, and literacy of emerging technologies.

#### Development of diversified measurement tools for SPOC-AIoT Teaching Model courses

The measurement tools developed in this study were designed for the subject of case study and module courses, not suitable to be popularized to different groups of students. Furthermore, it is not favorable for promoting emerging technologies as a permanent course. Hence, it is suggested to develop diversified measurement tools while promoting SPOC-AIoT Teaching Model courses to effectively measure the learning effects of students’ acceptance of SPOC-AIoT Teaching.

## Data availability statement

The raw data supporting the conclusions of this article will be made available by the authors, without undue reservation.

## Author contributions

C-CT and S-JL developed the theoretical formalism, performed the analytic calculations, and performed the numerical simulations. C-CC, C-CT, and Y-MC contributed to the final version of the manuscript. Y-MC and S-JL supervised the project. All authors contributed to the article and approved the submitted version.
